# Reply

**DOI:** 10.1016/j.jacig.2024.100332

**Published:** 2024-08-30

**Authors:** Maria Ödling, Niklas Andersson, Sandra Ekström, Niclas Roxhed, Jochen M. Schwenk, Sophia Björkander, Anna Bergström, Erik Melén, Inger Kull

**Affiliations:** a Department of Clinical Science and Education, Södersjukhuset, Karolinska Institutet, Stockholm, Sweden; b Institute of Environmental Medicine, Karolinska Institutet, Stockholm, Sweden; c Centre for Occupational and Environmental Medicine, Region Stockholm, Sweden; d Division of Micro and Nanosystems, KTH Royal Institute of Technology, Stockholm, Sweden; e MedTechLabs, Bioclinicum, Karolinska University Hospital, Solna, Sweden; f Science for Life Laboratory, Department of Protein Science, KTH Royal Institute Technology, Solna, Sweden; g Sachs’ Children and Youth Hospital, Stockholm, Sweden

To the Editor:

We thank Daungsupawong and Wiwanitkit for their comments^1^ on our work published in a recent issue of the *Journal of Allergy and Clinical Immunology: Global* and titled “COVID-19 vaccine uptake among young adults: Influence of asthma and sociodemographic factors.”^2^ In that study, we concluded that COVID-19 vaccine uptake among young adults is lower in individuals from households with lower socioeconomic status and among those with asthma, including uncontrolled asthma.

However, we would like to highlight the comment regarding vaccine data presented in our study, as the data were not self-reported. Information on vaccine uptake was obtained by linkage to the National Vaccination Register using personal identity numbers, with the Public Health Agency of Sweden responsible for the vaccination register. This register is mandatory, which means that all vaccinations against COVID-19 in Sweden are recorded.

## Disclosure statement

Supported by grants from the Swedish Research Council; the 10.13039/501100004359Swedish Research Council for 10.13039/100018696Health, Working Life, and Welfare; Formas; the Swedish Asthma and Allergy Research Foundation; the Swedish Heart-Lung Foundation; and Region Stockholm (the ALF project, and for cohort and database maintenance).

Disclosure of potential conflict of interest: E. Melén reports personal fees from 10.13039/100004325AstraZeneca, Chiesi, Sanofi, and Novartis outside the submitted work. The rest of the authors declare that they have no relevant conflicts of interest.
